# New findings of Pleistocene fossil turtles (Geoemydidae, Kinosternidae and Chelydridae) from Santa Elena Province, Ecuador

**DOI:** 10.7717/peerj.3215

**Published:** 2017-04-20

**Authors:** Edwin A. Cadena, Juan Abella, Maria D. Gregori

**Affiliations:** 1 Escuela de Ciencias Geológicas e Ingeniería, Yachay Tech, San Miguel de Urcuquí, Imbabura, Ecuador; 2 Facultad de Ciencias del Mar, Universidad Estatal de la Peninsula de Santa Elena, La Libertad, Santa Elena, Ecuador; 3 Universitat Autònoma de Barcelona, Institut Català de Paleontologia- Miquel Crusafont, Cerdanyola del Vallès, Spain

**Keywords:** Kinosternidae, Chelydridae, Testudines, Geoemydidae, Paleobiodiversity

## Abstract

New Pleistocene fossilized turtle remains from five localities of western Ecuador (Santa Elena Province) are described here. All these shell (carapace and plastron) fossil remains come from the Tablazo Formation and belong to three different lineages of cryptodires (“hidden-necked” turtles). The most abundant remains belong to geoemydids, attributed here to the genus *Rhinoclemmys* (indeterminate species). Less abundant in occurrence are the kinosternidids, attributed to *Kinosternon* (indeterminate species), and the first fossil record of chelydrids, *Chelydra*(indeterminate species), in the entirety of Central and South America.

## Introduction

**Figure 1:**
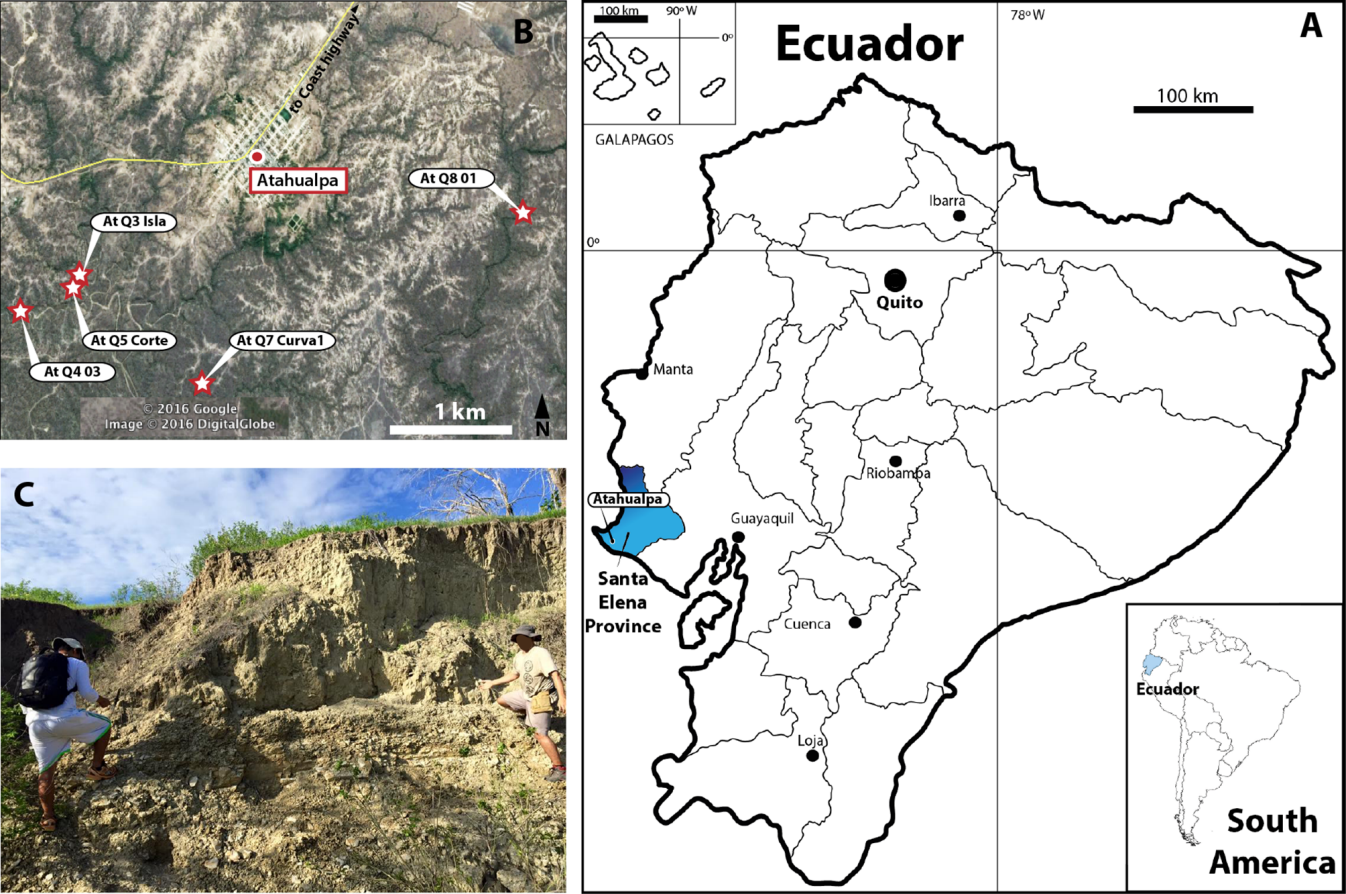
Map of Ecuador showing Santa Elena Province. (A) Santa Elena Province (blue in the map of Ecuador). (B) Atahualpa town and the geographical location of the five sites where the fossil materials described here were found. Satellite image taken from Google Earth, DigitalGlobe 2016. (C) One of the outcrops of Tablazo Formation at Atahualpa Isla locality (At. Q3 Isla).

Pleistocene fossil vertebrates from Santa Elena Province (western Ecuador) (Fig. 1A) constitute some of the most famous fossils of Ecuador, particularly by their excellent state of preservation, including specimens of extinct megafauna: giant sloths (*Eremotherium* and *Glossotherium)*, mastodons (*Stegomastodon*), and also medium-small in size groups of herbivorous and carnivorous mammals. Most of these fossils are found in asphalt seeps (tar pits) at four famous localities: La Carolina, Corralito, Rio Engabao, and the most recently described Tanque Loma (Hoffstetter, 1952; Edmund, 1965; Ficcarelli et al., 2003; Lindsey & Lopez, 2015). All these fossil localities make up part of the marine-related terraces known regionally as Tablazos, which have a complex and not yet fully resolved chronostratigraphic frame. This is also why recent works prefer to consider this entire sequence as Tablazo Formation (See Lindsey & Lopez, 2015).

The first fossil turtles from Santa Elena Province were reported by Hoffstetter (1952), attributing fragmentary material as belonging to *Testudo* sp. Later on, more fragmentary material of tortoises (*Geochelone* (*Chelonioidis*) cf. *gallardoi*) and geoemydids (*Geoemyda* sp.) were examined by Dr. Walter Auffenberg and reported in Edmund (1965). Also from the western margin of Ecuador, Pleistocene-Holocene turtle material represented by a partial epiplastron bone from a tortoise (*Geochelone* (*Chelonioidis*) sp.) was reported and figured in Cantalamessaa et al. (2001) from the San Jóse locality of Manabí Province. More fossil turtle remains from Santa Elena Province (Corralito and Rio Engabao localities) were collected during the eighties and nineties and have remained housed at the collections of the Royal Ontario Museum, in Toronto, Canada, material that it is not included in this study. Preliminary observations of part of this material were done by Carr (1991), indicating that at least two of the three extant *Rhinoclemmys* species inhabiting western Ecuador were represented in the fossils; however, no additional descriptions or studies have been done since then. This is also the case for all the Testudinidae (tortoises) material, which is currently under study by a student of the Pontificia Universidad Católica del Ecuador.

Here, we report and describe new material of fossil turtles (Geoemydidae, Kinosternidae and Chelydridae lineages) recently collected by J.A. as part of an extensive paleontological project being developed currently at the Universidad Estatal de la Peninsula de Santa Elena, La Libertad, Santa Elena Province, Ecuador (Fig. 1B). The fragmentary material of the shell (carapace and plastron) comes from five different fossil localities belonging to the Tablazo Formation (following Lindsey & Lopez, 2015) (Fig. 1C). Besides their taxonomical identification, we also discuss their paleobiogeographical implications.

## Materials and Methods

### Fossil material

The fossilized turtle material described here is housed in the paleontological collection at the Universidad Estatal de la Peninsula de Santa Elena (UPSE), La Libertad, Santa Elena Province, Ecuador. Comparisons of these fossils were done with extant representatives of each respective lineage as follows: Geoemydidae, all six species of *Rhinoclemmys* (see complete list of specimens in Cadena et al. (2012, Apx 1)), Kinosternidae, *Kinosternon: K. subrubrum* (MNHN 1976-10), *K. scorpioides* (ICN 7435, 7447, 7630), *K. leucostomum* (ICN 7443, 7677, MNHN 1956-35, NMW 1705), *K. bauri* (NMW 1692), and Chelydridae, *Chelydra serpentina* (MNHN 1870-465, ICN 6469, NMW 1749).

## Systematic Paleontology

**Table:** 

**Figure 2:**
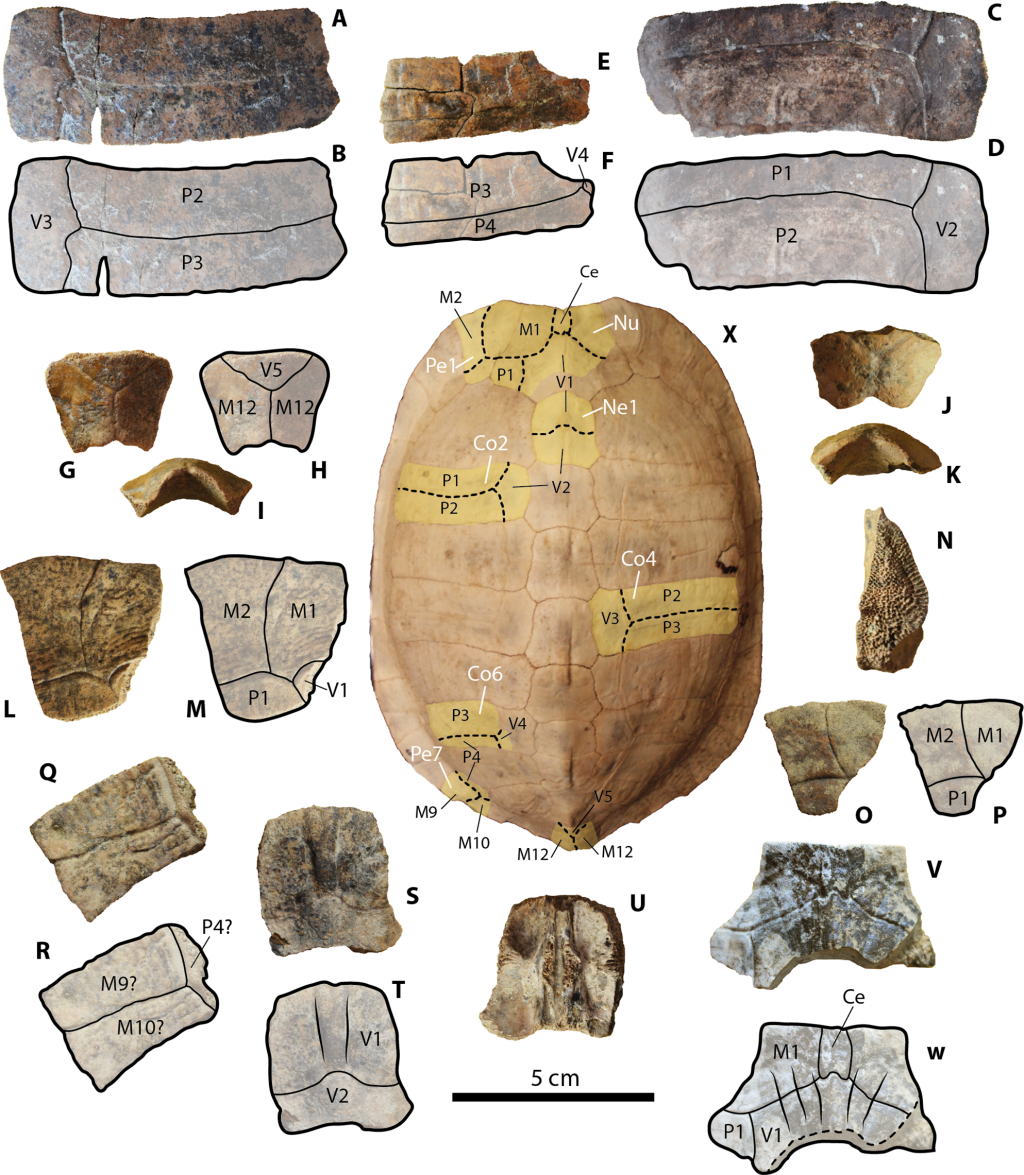
Geoemydid (*Rhinoclemmys.* sp. Indet.) carapacial material from Santa Elena Province. (A–B) UPSE-T0001 right costal 4 in dorsal view. (C–D) UPSE-T0031 left costal 2 in dorsal view. (E–F) UPSE-T0002 left costal 6 in dorsal view. (G–I) UPSE-T0012 posterior portion of pygal bone, (G–H) in dorsal view, (I) posterolateral view. (J–K) UPSE-T0013 posterior portion of pygal bone in dorsal view. (L–N) UPSE-T0015 left peripheral 1, (L–M) in dorsal view, (N) lateromedial view. (O–P) UPSE-T0016 left peripheral 1 in dorsal view. (Q–R) UPSE-T0019 left peripheral bone (potentially peripheral 7) in dorsal view. (S–U) UPSE-T0021 neural 1. (S–T) in dorsal view, (U) in ventral view. (V–W) UPSE-T0026 nuchal bone in dorsal view. (X) Carapace of the extant *Rhinoclemmys punctularia* CRI3706 in dorsal view, yellow shadows indicate similar bone described from Santa Elena in (A–W). Abbreviations: Ce, cervical scute; Co, costal bone; M, marginal scute; Nu, nuchal bone; P, pleural scute; Pe, peripheral bone; Py, pygal bone; V, vertebral scute.

**Figure 3:**
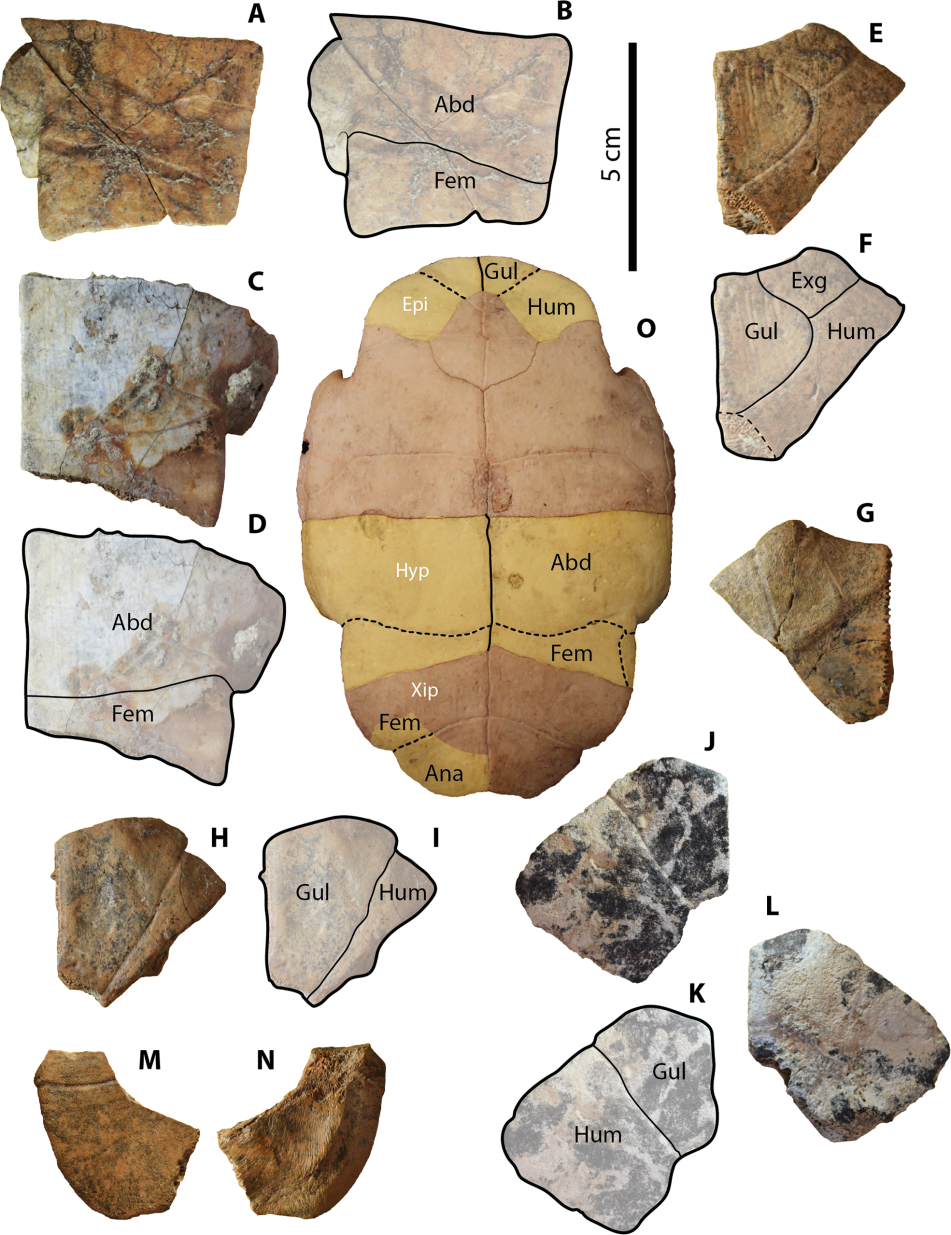
Geoemydid (*Rhinoclemmys.* sp. Indet.) plastral material from Santa Elena Province. (A–B) UPSE-T0006 right hypoplastron in ventral view. (C–D) UPSE-T0030 left hypoplastron in ventral view. (E–G) UPSE-T0009 left epiplastron. (E–F) in ventral view, (G) in dorsal view. (H–I) UPSE-T0008 left epiplastron in ventral view. (J–L) UPSE-T0035 right epiplastron. (J–K) in ventral view, (L) in dorsal view. (M–N) UPSE-T0010 right xiphiplastron in ventral (M) and dorsal view (N). (O) Plastron of the extant *Rhinoclemmys funerea* CRI4308 in ventral view, yellow shadows indicate similar bone described from Santa Elena in (A–N). Abbreviations: Abd, abdominal scute; Ana, anal scute; Epi, epiplastron bone; Exg, extra gular scute; Fem, femoral scute; Gul, gular scute; Hum, humeral scute; Hyp, hypoplastron bone; Xip, xiphiplastron bone.

Testudines Batsch, 1788
Cryptodira Cope, 1868
Geoemydidae Theobald, 1868
*Rhinoclemmys*Fitzinger, 1826
Sp. Indet. (Figs 2–3)

### Referred material and localities

Atahualpa Q3 “At. Isla” locality (2°19′17″S, 80°47′1″W), *Carapace*: UPSE-T0001, T0002, T0012, T0015, T0018, T0019, T0021. *Plastron*: UPSE-T0006, T0008, T0009, T0010. Atahualpa Q4 (2°19′28″S, 80°47′30″W), *Plastron*: UPSE-T0030. Atahualpa Q5 “At. Corte” locality (2°19′15″S, 80°47′16″W), *Carapace*: UPSE-T0026. Atahualpa Q7 “At. Curva” locality (2°19′17″S, 80°46′48″W), *Carapace*: UPSE-T0031. Atahualpa Q8 “Quebrada tiburón” locality (2o 18′59″S, 80o 45′10″W), *Plastron*: UPSE-T0035.

### Descriptions

***Carapacial bones.*** UPSE-T0001 corresponds to a right costal 4 (2.7 cm length, 7.3 cm width as preserved) (Figs. 2A–2B). It is almost rectangular in shape. As indicated by the sulcus, pleural 2 is slightly longer than pleural 3, supporting the attribution of the bone as right costal 4. The other potential anatomical attribution for this bone could be that it corresponds to a costal 2; however, the general trend between the pleurals on costal 2 bones in extant *Rhinoclemmys* species (see methods for list of specimens examined and *R. punctularia* CRI3706 shown at the center of Fig. 2) is to have a pleural 1 much shorter than pleural 2. Another characteristic of the sulcus between pleurals of UPSE-T0001 is to form a slightly curved to almost straight line, without clear development of indentations.

UPSE-T0031 is a left costal 2 (3.0 cm length, 8.2 cm width as preserved) (Figs. 2C–2D), having similar length medially versus laterally (almost rectangular in shape), with a pleural 1 much shorter than pleural 2 as indicated by the sulcus, supporting its attribution as left costal 2 (see previous paragraph). On its dorsal surface, square-shaped rings form the sculpturing pattern left by the pleural 1, which is less marked at the region covered by pleural 2.

UPSE-T0002 is a left costal 6 (2.3 cm length, 5.4 cm width as preserved) (Figs. 2E–2F), slightly longer laterally versus medially and with pleural 4 much longer than pleural 3 as indicated by the position of the sulcus between these two scutes. These two features support its attribution as left costal 6. On the most anterolateral margin (pleural 3 region), there is a sculpturing pattern of softly marked ridges running parallel to the length of the costal.

UPSE-T0012 and T0013 correspond to the most posterior portion of pygal bones, which are 2.3 cm length, 3 cm width as preserved (Figs. 2G–2I) and 1.6 cm length, 2.6 cm width as preserved (Figs. 2J–2K) respectively. On their dorsal surface both preserve the sulcus between vertebral 5 and marginal 12. Seen in lateral view (Figs. 2I–2K) both have a wide U-shaped notch at the posterior edge, slightly projected dorsally.

UPSE-T0015 and T0018 are left peripherals 1, which are 4.5 cm length, 4.3 cm width as preserved (Figs. 2L–2N) and 2.3 cm length, 2.6 cm width as preserved (Figs. 2O–2P), respectively. Both exhibit a well-preserved sulcus between marginals 1 and 2, and the sulcus between these two with pleural 1. UPSE-T0015 additionally shows that the vertebral 1 reached its most posteromedial corner. In lateral view, the sutural contact surface between the peripheral 1 and the nuchal is of densely packed horns with acute tips (Fig. 2N).

UPSE-T0019 is a left peripheral bone (potentially peripheral 7) (2.5 cm length, 3.6 cm width as preserved) (Figs. 2Q–2R). On its dorsal surface, the sulcus between marginals 9? and 10?, as well as of these two with pleural 4, is well preserved. A soft sculpturing pattern of wide separated ridges is also preserved on its dorsal surface.

UPSE-T0021 corresponds to a neural 1 (3.1 cm length, 2.7 cm width as preserved) (Figs. 2S–2U). On the dorsal surface, the sulcus between vertebrals 1 and 2 is well preserved, having a medial notch. Just after the notch there is a ridge that runs anteriorly towards the margin of the neural.

UPSE-T0026 is a nuchal bone, missing most of its right posterolateral and posteromedial portions (2.8 cm length, 4.5 cm width as preserved) (Figs. 2V–2W). On the dorsal surface, the shape of the cervical scute is indicated by the sulcus, suggesting that it was rectangular in shape and restricted between marginal 1 and vertebral 1. This last scute was anterolaterally restricted to the nuchal, without reaching the peripherals.

***Plastral bones***. UPSE-T0006 and T0030 are right and left hypoplastra, which are 4.2 cm length, 5.8 cm width as preserved (Figs. 3A–3B) and 6.1 cm length, 6.5 cm width as preserved (Figs. 3C–3D), respectively. On their ventral surface, both exhibit a well-defined abdomino-femoral sulcus, having the medial length of the femoral scute twice shorter than at its lateral margin.

UPSE-T0009 is a left epiplastron (4.2 cm length, 3.8 cm width as preserved) (Figs. 3E–3G). On its ventral surface, the gular scute (as indicated by the sulci) is divided in two, creating an extragular. However, in the absence of the right epiplastron, it is not possible to establish if this particular arrangement of the gular and extragular was a pathological anomaly or if it really was a distinct feature of this individual occurring symmetrically in both epiplastra. On the dorsal surface of the epiplastron (Fig. 3G), there is evidence that the extragular scute reached the step before the visceral surface of the bone.

UPSE-T0008 and T0035 are the left (missing the posterolateral portion) and right epiplastra, which are 3.1 cm length, 3.2 cm width as preserved (Figs. 3H–3I) and 5.6 cm length, 4.9 cm width as preserved (Figs. 3J–3L), respectively. Both epiplastra exhibit an equal pattern of gular and humeral scutes as indicated by their sulci, with the gular scute reaching the posteromedial edge of the epiplastron. Posteromedially, the gular reached the most anterior portion of the entoplastron.

UPSE-T0010 is a right xiphiplastron (3.1 cm length, 2.9 cm width as preserved) (Figs. 3M–3N) missing most of its anteromedial portion. On its ventral surface, part of the femoro-anal sulcus is visible. On the dorsal surface there is a narrow depression, followed by a ridge that marks the separation between the visceral and the marginal surfaces of the bone. A specimen of *Rhinoclemmys funerea* CRI4308 (Fig. 3O) is used for anatomical visualization of the fossil elements described from Santa Elena.

**Table:** 

**Figure 4:**
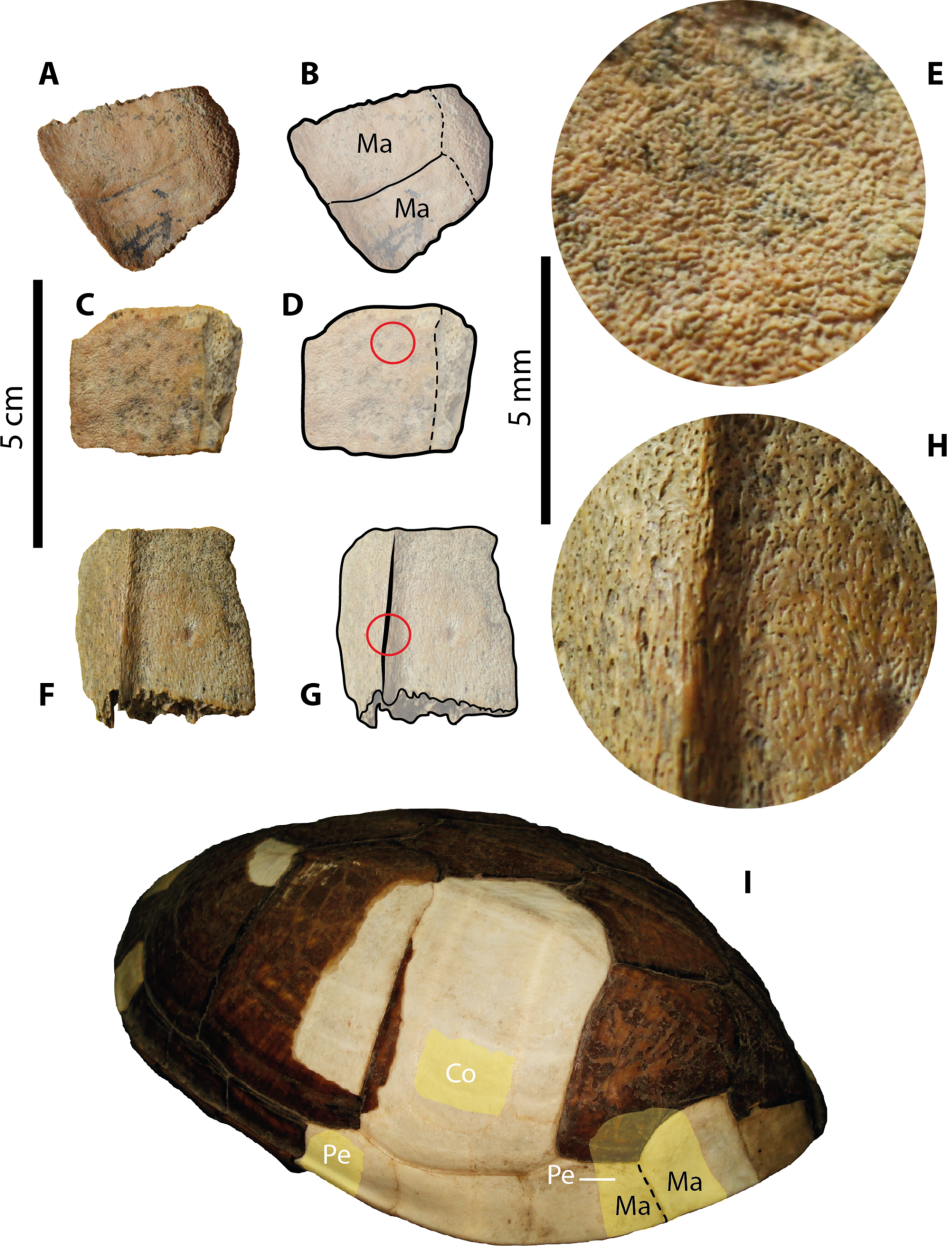
Kinosternid (*Kinosternon.* sp. Indet.) carapacial material from Santa Elena Province. (A–B) UPSE-T0022 peripheral bone in dorsal view. (C–E) UPSE-T0023 partial peripheral bone. (C–D) in dorsal view. (E) close-up of the red circle in (D) showing the microvermiculation sculpturing pattern. (F–H) UPSE-T0025 peripheral bone from the bridge region. (F–G) in dorsal view, (H) close-up of the red circle in (G) showing the sculpturing pattern. (I) Carapace of the extant *Kinosternon scorpioides* ICN 7435 in left-dorsolateral view, yellow shadows indicate similar bone described from Santa Elena in (A–G). Abbreviations: Co, costal bone; Ma, marginal scute; Pe, peripheral bone.

Kinosternidae *Agassiz, 1857*
*Kinosternon*Spix, 1824
Sp. Indet. (Fig. 4)

### Referred material and locality

Atahualpa Isla locality, *Carapace*: UPSE-T0022, T0023 and T0025.

### Descriptions

***Carapacial bones***. UPSE-T0022 corresponds to a peripheral bone from the posterior margin of the carapace (1.6 cm length, 1.5 cm width as preserved) (Figs. 4A–4B), having a very dense-pitted microvermiculation sculpturing pattern in both dorsal and ventral surfaces of the bone. The sulcus between marginals is slightly visible on the dorsal surface of the bone, and although its contact with the pleural is not well preserved, it is clear that the pleural reached the most medial portion of the bone. The most lateral edge of the bone is slightly facing upwards.

UPSE-T0023 is a portion of a costal bone (1.8 cm length, 1.5 cm width as preserved) (Figs. 4C–4E). The sculpturing pattern is of dense-pitted microvermiculation (Fig. 4E), similar to UPSE-T0022. The average thickness of the bone is 7 mm.

UPSE-T0025 is a nearly complete peripheral bone from the bridge region (1.7 cm length, 1.6 cm width as preserved) (Figs. 4F–4H), missing the most medial margin. The sculpturing pattern is the same as the one described for UPSE-T0022 and T0023 (Fig. 4H).

**Table:** 

**Figure 5:**
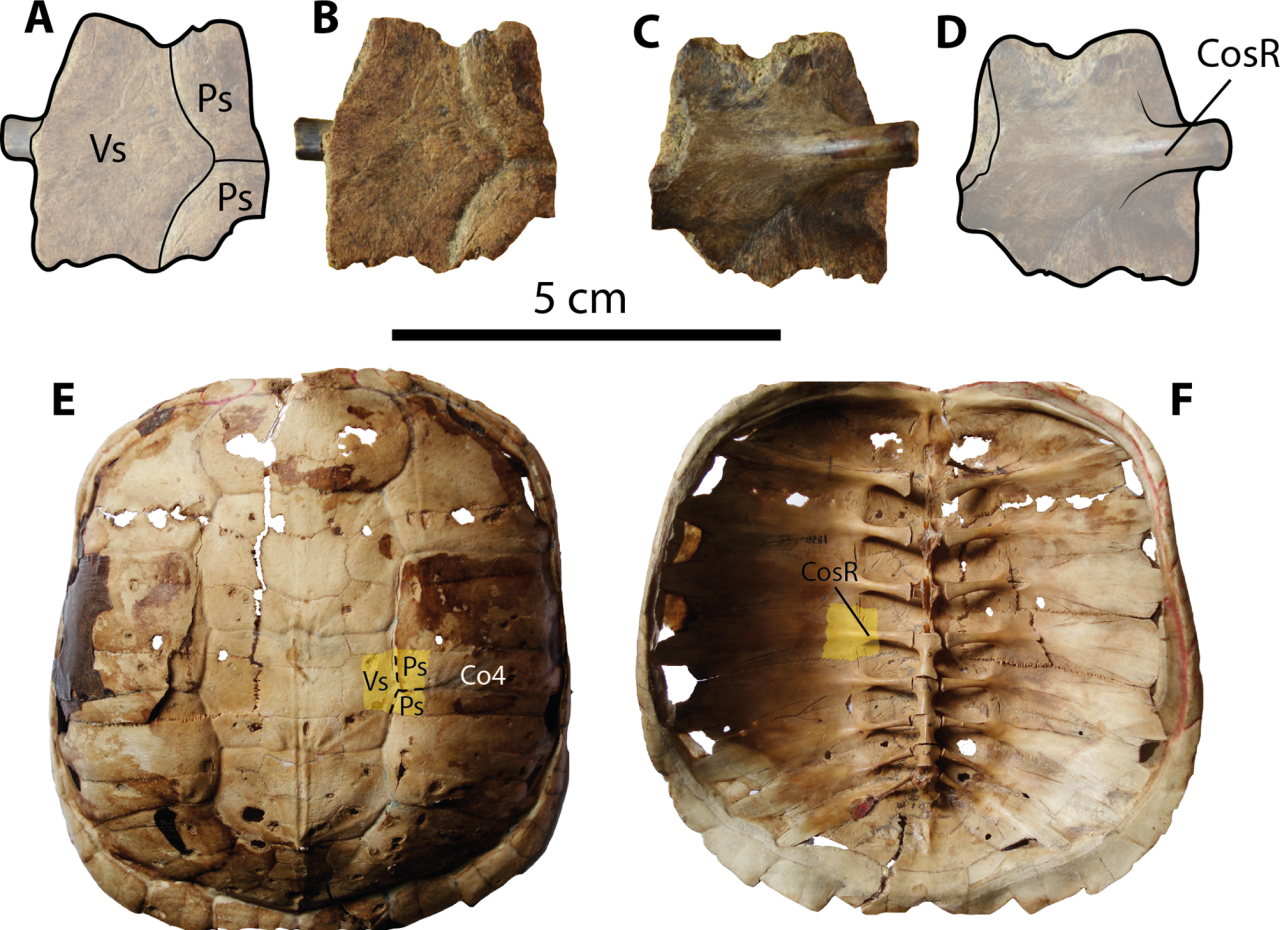
Chelydrid (*Chelydra.* sp. Indet.) partial costal bone from Santa Elena Province. (A–D) UPSE-T0003 medial portion of a costal bone. (A–B) in dorsal view, (C–D) in ventral view. (E–F) Carapace of the extant *Chelydra serpentina* MNHN 1870-1165, (E) carapace in dorsal view, (F) carpace in ventral view, yellow shadows indicate similar bone described from Santa Elena. Abbreviations: Co, costal bone; CosR, costal rib; Ps, pleural scute; Vs, vertebral scute.

Chelydridae Gray, 1831
*Chelydra Schweigger, 1812*
Sp. Indet. (Fig. 5)

### Referred material and locality

Atahualpa Isla locality, *Carapace*: UPSE-T0003

### Description

***Carapacial bone***. UPSE-T0003 corresponds to a medial portion of a costal bone (potentially right costal 4) (1.9 cm length, 2 cm width as preserved) (Figs. 5A–5D). On its dorsal surface, the sulcus between pleural scutes is visible, as well as the sulcus between these two and the vertebral. On its ventral surface, part of the dorsal rib that articulates medially with the thoracic vertebra is well defined, indicating that there was a costovertebral tunnel formed by the medial processes of costal ribs and the thoracic vertebrae.

## Discussion

### Comparisons and taxonomical attributions

#### Rhinoclemmys assignation

**Figure 6:**
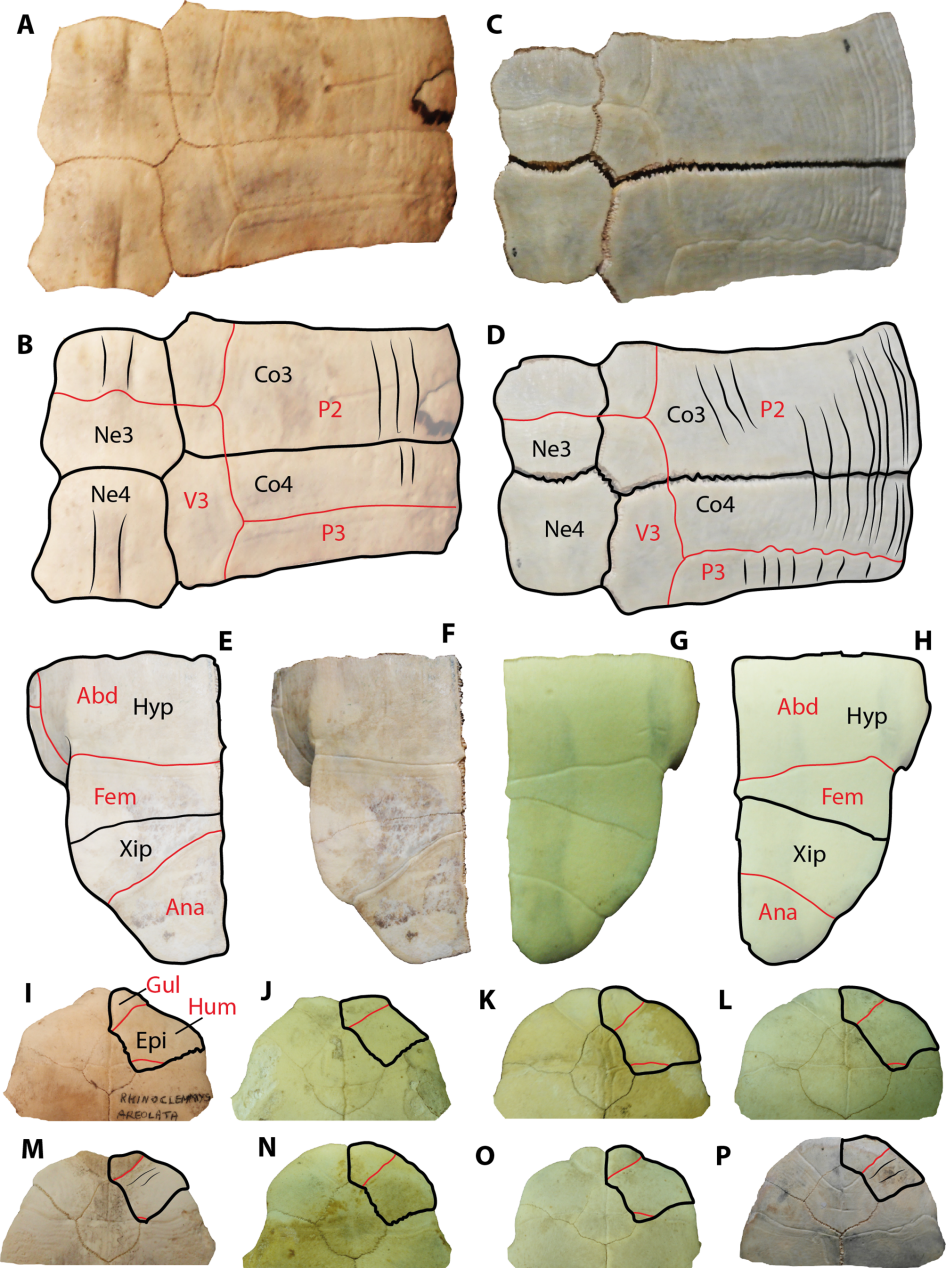
Extant representatives of geoemydids and emydids used for comparisons with the fossil material from Santa Elena Province. (A–B) *Rhinoclemmys punctularia* CRI3706 (Chelonian Research Institute, Florida, USA) neurals 3 and 4, right costal 3 and 4 in dorsal view. (C–D) *Trachemys scripta* MTKD 26593 (Senckenberg Museum of Natural History, Dresden collections, Germany) neurals 3 and 4, right costal 3 and 4 in dorsal view. (E–F) *Trachemys scripta* MTKD 26593, right hypo and xiphiplastron in ventral view. (G–H) *Rhinoclemmys annulata* CRI0048, right hypo and xiphiplastron in ventral view Abbreviations: Abd, abdominal scute; Ana, anal scute; Co, costal bone; Hyp, hypoplastron; Ne, neural bone; P, pleural scute; V, vertebral scute; Xip, xiphiplastron.

Besides tortoises, the geoemydids are the most abundant fossilized turtle remains found in Santa Elena Province, which is in the southwestern corner of Ecuador. In particular, the geoemydid material described herein resembles shell bone elements of representatives from the extant genus *Rhinoclemmys* by the following features: costal bones with smooth to weak sculpturing pattern of annuli on their dorsal surface (mostly restricted to the lateral portions), as well as straight line sulcus between pleural scutes (Figs. 6A–6B). In these aspects, geoemydids differ from emydids, particularly from representatives of *Trachemys,* which generally exhibit a more deeply-marked sculpturing pattern developed along almost the entire surface of costals and a much wavier sulcus between pleurals (Figs. 6C–6D). The shape and morphology of neural bones of geoemydids described herein is also the same as in extant representatives of *Rhinoclemmys* (Fig. 2X), developing a ridge anteriorly to the sulcus between vertebral scutes (Figs. 6A–6B). In contrast, the neurals of emydids, particularly in *Trachemys scripta*, lack a ridge (Figs. 6C–6B). Another morphological characteristic that allows the ability to distinguish the extant or fossil members of the *Rhinoclemmys* spp. as the one described herein from *Trachemys* spp. is the narrower medial length of the femoral scutes, which is longer in *Trachemys scripta* for example (Figs. 6E–6H).

Extant representatives of *Rhinoclemmys* commonly exhibit a medially wider and a weaker marked axillary buttress scar (Supplemental Information 1), in contrast to extant representatives of *Trachemys*, particularly *Trachemys scripta* for which the most common condition is to have a narrow, elongated, and deeply-marked axillary buttress on the ventral surface of costal 1.

In terms of the plastral bone elements described herein and assigned as belonging to *Rhinoclemmys* sp. (UPSE-T0008, T0009 and T0035, see Fig. 3), we want to point out that although they all exhibit differences in the scutes pattern and shape of the epiplastron as to potentially be considered to belong to three different taxa, we refrain from a taxonomical splitting. The main reasons to avoid this splitting are the fragmentary nature of the material and the absence of their opposite corresponding element (i.e the left epiplastron for the right one), which could lead us to believe that the scutes pattern is symmetrical in both epiplastra and does not correspond to potential pathological anomalies. It seems plausible, as it was initially suggested by Carr (1991) that more than one taxa of *Rhinoclemmys* inhabited the southwestern edge of Ecuador during the Pleistocene.

A comparison between the anterior plastral lobe, including the outline of the right epiplastron and its scutes pattern for the extant species of *Rhinoclemmys*, is shown in Figs. 6I–6O, and it is used here to show that the most common condition in this genus is the lack of strongly-marked annuli on the ventral surface of the bone, in contrast to representatives of *Trachemys*, particularly *T. scripta*, which exhibit a more strongly marked annuli.

#### Kinosternids assignation

The fragmentary material of kinosternids from Santa Elena Province described herein (UPSE-T0022, T0023 and T0025) is characterized by their densely microvermiculated sculpture and bridge peripherals, developing a narrow ridge along their most dorsolateral edges, which are similar features exhibited by extant and fossil species of *Kinosternon* genus from North and South America (Cadena, Jaramillo & Páramo-Fonseca, 2007; Bourque, 2015). The relative size as well as the thickness of the bone elements attributed herein to *Kinosternon* sp. are also in agreement with similar bone elements of extant representatives of this genus, including also, for example, the upward facing of the most lateral margin of some of the posterior peripherals (see Fig. 4I). We ruled out that this material belongs to chelydrid elements, not only by the characteristics already mentioned, but also by considering that the posterior carapacial peripherals of chelydrids develop lateral margin dentations, which are absent in UPSE-T0022. Also, we ruled out that belongs to immature testudinids, because the sulcus between marginals in UPSE-T0022 lacks of the typical condition present in *Chelonoidis* (the extant and fossil South American tortoises), which is of a deep sulcus with double-wall shape, and almost overlapping the sutural contact between costal and peripheral bones, particularly in peripherals from the posterior margin of the carapace (Supplemental Information 2).

#### Chelydrids assignation

The chelydrid material described here (UPSE-T0003, Fig. 5) resembles representatives of the extant genus *Chelydra* (Figs. 5E–5F) in the following characteristics: a thin thickness of the bone (7 mm thick) for a juvenile-adult stage and the development of a costovertebral tunnel between costal ribs and thoracic vertebrae. Although the chelydrid material from Atahualpa described herein cannot be attributed to any species in particular, it could be related to the extant species *Ch. acutirostris*, which currently inhabits the tropical western margin of South America and part of Central America (Turtle Taxonomy Working Group, 2014). The chelydrid material from Santa Elena Province also represents the first fossil record of this lineage in Central and South America. Unfortunately, the deficiency of a well-calibrated age for the Tablazo Formation deposits makes it difficult to pinpoint an exact age. Therefore, this and all other turtles described herein can only be attributed as Pleistocene (possibly late Pleistocene) in age, based mostly on stratigraphical correlations with the other fossil localities in the area, which have been recently summarized by Lindsey & Lopez (2015).

### Paleobiogeographical implications

Despite the lack of a precise chronostratigraphical framework for the fossil turtle material described here, it is evident that at least one of the three lineages occurring at the Atahualpa localities (Geoemydidae) has suffered either reductions in their geographical distribution or local extinctions, potentially during the very late Pleistocene and the Holocene. Currently, there is one species (*Rhinoclemmys melanosterma*) that is still inhabiting some regions of the west coast of Ecuador (Esmeraldas and Manabí Provinces) (Torres-Carvajal et al., 2015). However, they have no occurrence today at Santa Elena Province or the northwest of Peru where they used to live during the Pleistocene (Seymour, 2015, and this study). Chelydrids (*Chelydra acutirostris* species) and kinosternids (*Kinosternon leucostomum* species) are also lacking in appearance today in Santa Elena Province. They are still, however, inhabiting some regions of the neighboring Guayas and Manabí Provinces (Torres-Carvajal et al., 2015). The causes for the reduction and/or local extinction of these lineages of turtles in the west coast of Ecuador and Peru are difficult to establish and probably correspond to a combination of factors, including an increase in arid conditions of these regions and potential ecological effects caused by human or other faunal components.

The Pleistocene record of chelydrids and kinosternids presented herein constitute the first fossil record for South America and the first one for the western margin of the continent, respectively. Together with the previously reported record of geoemydids from Talara, Perú (Seymour, 2015), these records show that the three lineages, since their arrival to South America probably during the final formation of the Isthmus of Panama, around 2.8 Ma (sensu O’Dea et al., 2016), spread and inhabited the western margin of the continent until at least the late Pleistocene (0.01 Ma), with a more recent (Holocene) reduction/local extinction in this region.

##  Supplemental Information

10.7717/peerj.3215/supp-1 Supplemental Information 1 Anterior left portion of the carapace in ventral view for some extant species of *Rhinoclemmys* (A) *R. annulata* CRI 3638. (B) *R. areolata* CRI 8318. (C) *R. diademata* CRI 1339. (D) *R. diademata* CRI 1516. (E) *R. pulcherrima* CRI 1438. (F) *R. punctularia* CRI 2514. Green shadow represents the axillary buttress scar.

10.7717/peerj.3215/supp-2 Supplemental Information 2 Posterior portion of carapace of some extant South American *Chelonoidis* species First row. *C. carbonaria* ICN7619. Second row, *C. denticulata* MTKD48038 (juvenil specimen). Third row, *C. carbonaria* MTKD42484 posterior view of the carapace, showing clearly the deep and double-wall shaped sulci. Fourth row, *C. carbonaria* MTKD3821, exhibiting a sulcus between marginals recaching the peripherals-costars sutural contact. Sulcus between marginals in red, sutural contact between peripherals in black.

## Institutional abbreviations

ICN: Instituto de Ciencias Naturales, Universidad Nacional de Colombia, Bogotá, Colombia
MNHN: Muséum National d’Historie Naturelle, Paris, France
MTKD: Senckenberg Museum of Natural History, Dresden collections, Germany
NMW: Natural History Museum of Vienna, Austria
UF: University of Florida, Herpetology Collection, Gainesville, Florida, USA
UPSE: paleontological collection, Universidad Estatal de la Peninsula de Santa Elena La Libertad, Santa Elena Province, Ecuador

## Additional Information and Declarations

### Data Availability

The following information was supplied regarding data availability: The raw data is included in the figures in the manuscript.
