# Supplementary figures and images for: New findings of Pleistocene fossil turtles (Geoemydidae, Kinosternidae and Chelydridae) from Santa Elena Province, Ecuador
Source: PeerJ. 2017 Apr 20;5:e3215. doi: 10.7717/peerj.3215 (PMC5401626; doi:10.7717/peerj.3215)

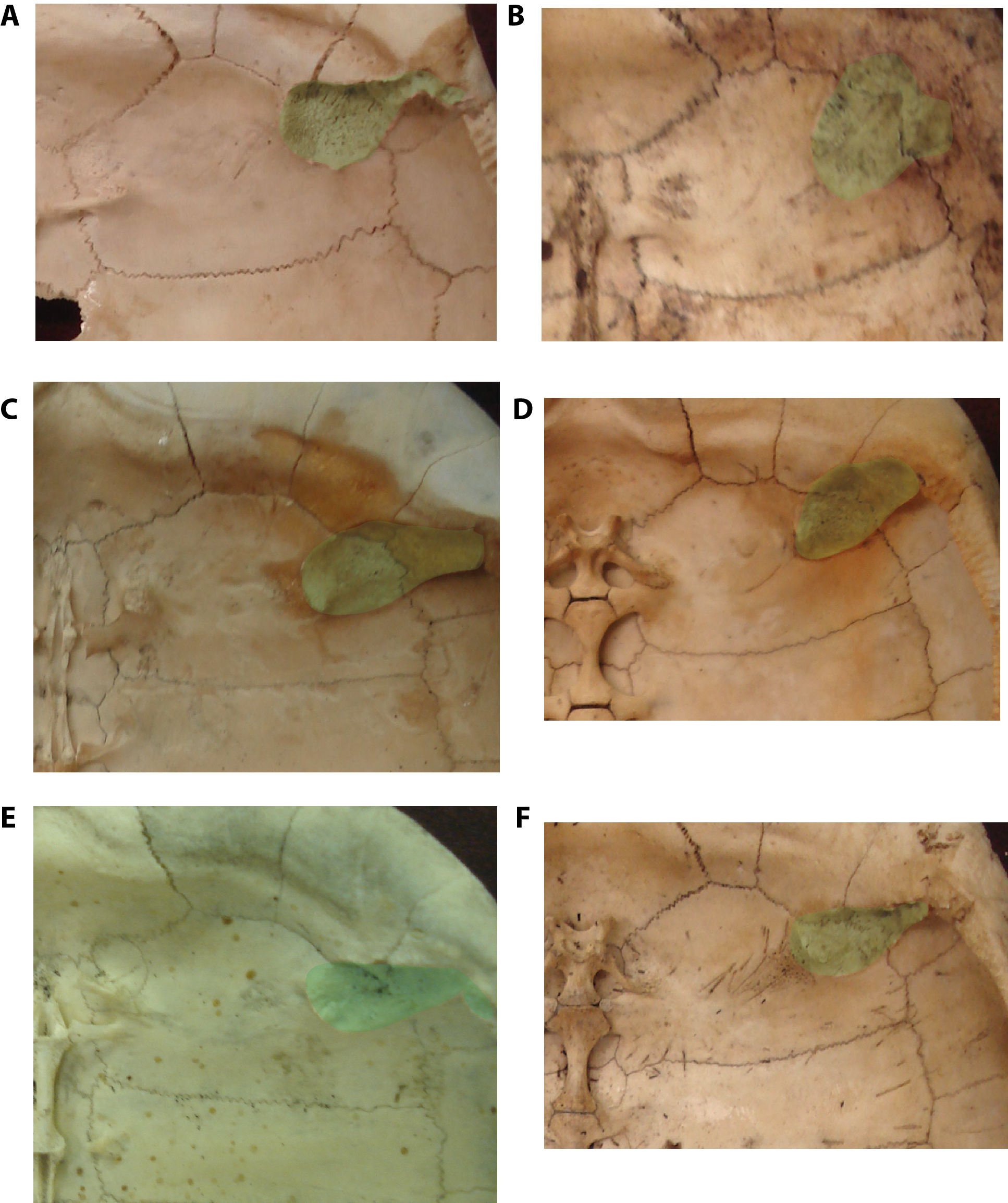

Supplement: Supplemental Information 1 — (A) R. annulata CRI 3638. (B) R. areolata CRI 8318. (C) R. diademata CRI 1339. (D) R. diademata CRI 1516. (E) R. pulcherrima CRI 1438. (F) R. punctularia CRI 2514. Green shadow represents the axillary buttress scar. [file peerj-05-3215-s001.png]

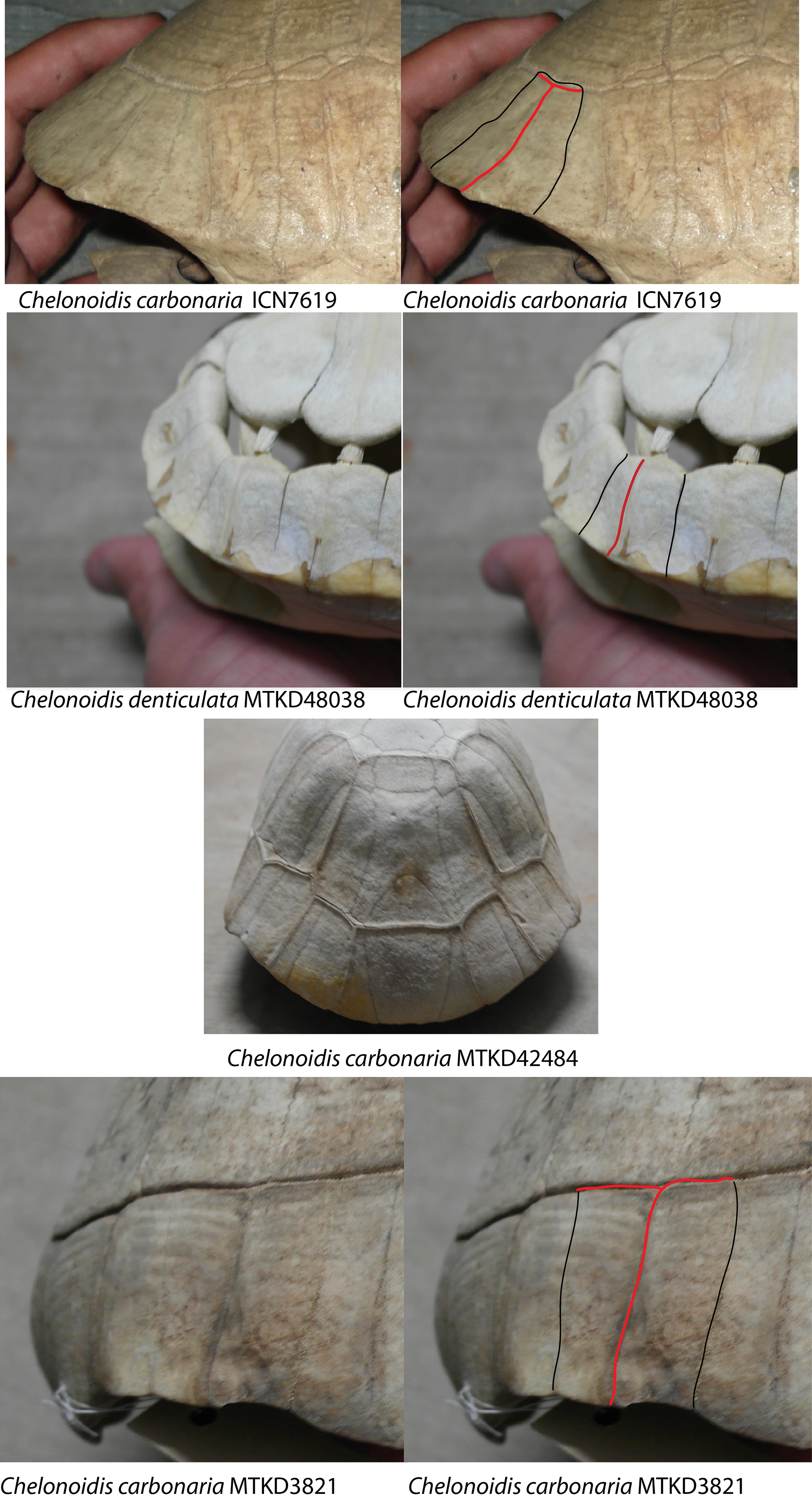

Supplement: Supplemental Information 2 — First row. C. carbonaria ICN7619. Second row, C. denticulata MTKD48038 (juvenil specimen). Third row, C. carbonaria MTKD42484 posterior view of the carapace, showing clearly the deep and double-wall shaped sulci. Fourth row, C. carbonaria MTKD3821, exhibiting a sulcus between marginals recaching the peripherals-costars sutural contact. Sulcus between marginals in red, sutural contact between peripherals in black. [file peerj-05-3215-s002.png]
